# Supplementary material for: Evaluation of Formalin-Inactivated Vaccine Efficacy against Red Seabream Iridovirus (RSIV) in Laboratory and Field Conditions
Source: Vaccines (Basel). 2024 Jun 19;12(6):680. doi: 10.3390/vaccines12060680 (PMC11209460; doi:10.3390/vaccines12060680)
Supplement: Supplementary file 1 [file vaccines-12-00680-s001.zip › vaccines-3049678-supplementary.pdf]

vaccines

MDPI

Supplemental Table S1. Efficacy of formalin-inactivated RSIV vaccine in rock bream under laboratory and field conditions. The table shows the results of vaccine efficacy in two different commercial farms comparing vaccinated and control groups across different sampling points (weeks post-vaccination).

Experimental condition		Sampling point (weeks post-vaccination)	Group	Average body weight (g)	No. of tested fish	No. of survival fish	Cumulative mortality (%)	Relative percentage of survival (RPS, %)
Farm A	Artificially infected[a]	3	Vaccinated	21.4 ± 4.0	15	9	40.0	60.0
	(In laboratory conditions)	3	Control	17.1 ± 1.7	15	0	100.0	-
		6	Vaccinated	53.4 ± 15.9	11	7	36.4	63.6
		6	Control	53.8 ± 12.3	12	0	100.0	-
		9	Vaccinated	88.8 ± 16.9	12	8	33.3	66.7
		9	Control	67.6 ± 9.8	15	0	100.0	-
		12	Vaccinated	130.3 ± 19.5	19	15	21.1	78.9
		12	Control	121.9 ± 10.0	20	0	100.0	-
		15	Vaccinated	130.3 ± 19.5	10	8	20.0	80.0
		15	Control	121.9 ± 10.0	13	0	100.0	-
		18[c]	Vaccinated	152.7 ± 24.5	16	12	25.0	75.0
		18[c]	Control	147.6 ± 18.2	18	0	100.0	-
	Non-infected	18[c]	Vaccinated	152.7 ± 24.5	31,100[d]	30,450[e]	2.09	-
	(In field conditions)	18[c]	Control	147.6 ± 18.2	10,400[d]	10,080[e]	3.08	-
Farm B	Artificially infected[a]	3	Vaccinated	50.9 ± 7.3	15	10	33.3	66.7
	(In laboratory conditions)	3	Control	55.7 ± 5.1	15	0	100.0	-
	Naturally infected[b]	12[c]	Vaccinated	88.5 ± 14.5	30,700[d]	19,520[e]	36.43	54.67
	(In field conditions)	12[c]	Control	79.5 ± 9.0	31,000[d]	6,100[e]	80.32	-

[a] Rock bream artificially infected with intraperitoneal injection of IVS-1 (10^4 copies/mL) at 25°C under laboratory conditions.
[b] Rock bream naturally infected with RSIV under field conditions.
[c] At the ends of the field trial, fish were either transported to the laboratory for vaccine efficacy analysis or evaluated on-site.
[d] Initial number of fishes stocked in the marine cage.
[e] Number of fishes surviving in the marine cage at the end of the field trial.
